# In Silico Characterization of miRNA and Long Non-Coding RNA Interplay in Multiple Myeloma

**DOI:** 10.3390/genes7120107

**Published:** 2016-11-29

**Authors:** Domenica Ronchetti, Martina Manzoni, Katia Todoerti, Antonino Neri, Luca Agnelli

**Affiliations:** 1 Department of Oncology and Hemato-Oncology, University of Milano, 20122 Milan, Italy; domenica.ronchetti@unimi.it (D.R.); martina.manzoni@unimi.it (M.M.); 2 Hematology Unit, Fondazione IRCCS Ca′ Granda, Ospedale Maggiore Policlinico, 20122 Milan, Italy; 3 Laboratory of Pre-Clinical and Translational Research, IRCCS-CROB, Referral Cancer Center of Basilicata, 85028 Rionero in Vulture (PZ), Italy; katia.todoerti@gmail.com

**Keywords:** multiple myeloma, lncRNA, miRNA

## Abstract

The identification of deregulated microRNAs (miRNAs) and long non-coding RNAs (lncRNAs) in multiple myeloma (MM) has progressively added a further level of complexity to MM biology. In addition, the cross-regulation between lncRNAs and miRNAs has begun to emerge, and theoretical and experimental studies have demonstrated the competing endogenous RNA (ceRNA) activity of lncRNAs as natural miRNA decoys in pathophysiological conditions, including cancer. Currently, information concerning lncRNA and miRNA interplay in MM is virtually absent. Herein, we investigated in silico the lncRNA and miRNA relationship in a representative datasets encompassing 95 MM and 30 plasma cell leukemia patients at diagnosis and in four normal controls, whose expression profiles were generated by a custom annotation pipeline to detect specific lncRNAs. We applied target prediction analysis based on miRanda and RNA22 algorithms to 235 lncRNAs and 459 miRNAs selected with a potential pivotal role in the pathology of MM. Among pairs that showed a significant correlation between lncRNA and miRNA expression levels, we identified 11 lncRNA–miRNA relationships suggestive of a novel ceRNA network with relevance in MM.

## 1. Introduction

Multiple myeloma (MM) is a malignant proliferation of bone marrow (BM) plasma cells (PCs) characterized by a highly heterogeneous genetic background and clinical course, ranging from pre-malignant monoclonal gammopathy of undetermined significance to smoldering MM, symptomatic MM, and plasma cell leukemia (PCL). Approximately half of MM tumors present a hyperdiploid karyotype generally in association with a better prognosis, whereas non-hyperdiploid tumors are enriched in primary IGH translocations events [1]. The identification of overexpressed microRNAs (miRNAs) in MM has undoubtedly contributed to MM molecular classification. Specific miRNAs have been identified as deregulated in distinct subgroups of MM patients, mainly in association with IGH translocations or allelic imbalances, and individual miRNAs have been demonstrated to be involved in neoplastic transformation and the progression of the disease [2,3,4,5,6,7].

miRNAs can regulate gene expression at the post-transcriptional level through binding to miRNA response elements (MREs) in the untranslated regions or the coding sequence of target genes [8]. It has been shown that different RNA molecules harboring MREs can compete for a common pool of miRNAs, thus acting as competing endogenous RNAs (ceRNAs) [9,10]. There is increasing evidence that ceRNA crosstalk occurs widely in cellular processes, and its perturbation will unsettle the transcriptomic equilibrium leading to disease initiation and progression [10,11]. The miRNA-mediated circuits among transcripts can involve coding as well as noncoding transcripts, including long noncoding RNAs (lncRNAs), which are ncRNAs larger than 200 nucleotides in length [12,13,14]. lncRNAs act as essential components of complex gene regulatory network by regulating gene expression at the transcriptional, post-transcriptional, and epigenetic levels [15,16]. Thousands of lncRNAs have been annotated in eukaryotic genomes (annotation from LncRNAdb, Ensembl, Broad Institute, Gencode 13, Refseq, and NONCODE collected in LNCipedia repository [17]), many of which are preferentially located in the cytoplasm [18], where they could be engaged in miRNA-mediated interactions with other transcripts. Both computational and experimental evidence support the extensive targeting of lncRNAs by miRNAs [19]. Recent studies have demonstrated the ceRNA activity of lncRNAs as natural miRNA decoys in human development and pathophysiological conditions [20]. Systematic analyses of lncRNA-associated ceRNA network have been performed in breast cancer [21,22], gastric cancer [23], and glioblastoma multiforme [24]. Recently, we and others have described the pattern of lncRNAs deregulation in MM distinct subgroups [25] and in human MM cell lines resistant to proteasome inhibitors [26]; however, a portrait of lncRNA and miRNA interplay in the pathology is still lacking.

The present study was therefore aimed at investigating in silico the lncRNA and miRNA relationship in a cohort of MM patients and in normal BM PCs. We searched for putative significant correlation between lncRNAs and miRNAs expression level in paired samples, with the support of target prediction analysis. Overall, our findings identified lncRNA–miRNA pairs suggestive of a novel ceRNA network with a potential impact in MM biology.

## 2. Materials and Methods

### 2.1. Patients

The study included bone marrow aspirates from newly diagnosed 95 MM and 30 PCL patients obtained during standard diagnostic procedures at the IRCCS Institution in Milan, as previously reported [3]. Patients employed for the study were representative of the major molecular characteristics of the disease. Samples were characterized for the presence of the most frequent chromosomal translocations and the ploidy status based on fluorescence in situ hybridization (FISH) evaluation criteria, as previously described [27]. Written informed consent was obtained from all patients in accordance with the declaration of Helsinki. Four normal controls were purchased from Voden, Medical Instruments IT.

### 2.2. Expression Profiling

miRNA and lncRNA expression data was available for 95MM, 30 PCL, and 4 normal samples (GSE87830). Normal sample RNAs were collected from four normal bone marrow donors (Voden, Medical Instruments IT, Meda, Milan, Italy). miRNA profiling was generated using GeneChip^®^ miRNA 3.0 Array (Affymetrix Inc., Santa Clara, CA, USA) as previously described [3]. lncRNAs expression data of the paired samples were extracted from CEL files generated on GeneChip^®^ Gene 1.0 ST Array (Affymetrix Inc., Santa Clara, CA, USA) as previously reported [28]. Briefly, expression data have been normalized using an RMA procedure at the probe cluster ID annotation level. Cross-hybridization probes were filtered out. We combined annotated probes with both Ensembl transcripts (GRCh37/hg19 assembly, http://grch37.ensembl.org/index.html) and lncRNAs from the version 4.0 of the LNCipedia repository database (http://www.lncipedia.org/), based on the chromosome localization of the target sequence identified by each probe; afterwards, we considered only probes univocally referable to lncRNA transcripts (i.e., that do not overlap with Ensembl transcripts). To summarize probes related to each lncRNA, we considered their median expression value [25]. The differentially expressed miRNAs and lncRNAs discriminating MM PCs and normal PC counterparts were identified with Significant Analysis of Microarrays (SAM) software version 5.00 [29] using the web application provided in the shiny package for R software [30].

### 2.3. Statistical Analysis

A Wilcoxon rank-sum test was applied using standard functions in the R base package. The Benjamini–Hochberg method was applied for multiple testing correction. miRNA targets custom predictions were obtained for lncRNA sequences by merging the results of two different algorithms: the RNA-22 version 2.0 prediction algorithm [31] and miRanda [32], which both allow customizing input sequences and parameters. The RNA-22 perl script was run on the combination of the 459 miRNA and 1546 (number of different transcripts corresponding to 235 lncRNAs) lncRNA FASTA sequences selected as relevant among all miRNAs and lncRNAs respectively detected by the arrays. Default sensitivity/specificity ratio of 1.032 was chosen, provided a minimum seed size of 7 bases with only one mismatch exception, a minimum number of 12 paired-up bases in heteroduplex with -12 Kcal/mol maximum folding energy, and no more than one G-U wobble in the seed region. The miRanda algorithm was run under default conditions, without any a priori restrictions on score, energy, or trimming parameters.

## 3. Results

### 3.1. lncRNA and miRNA Interplay in MM

#### 3.1.1. Selection of Relevant lncRNAs and miRNAs

**Figure 1:**
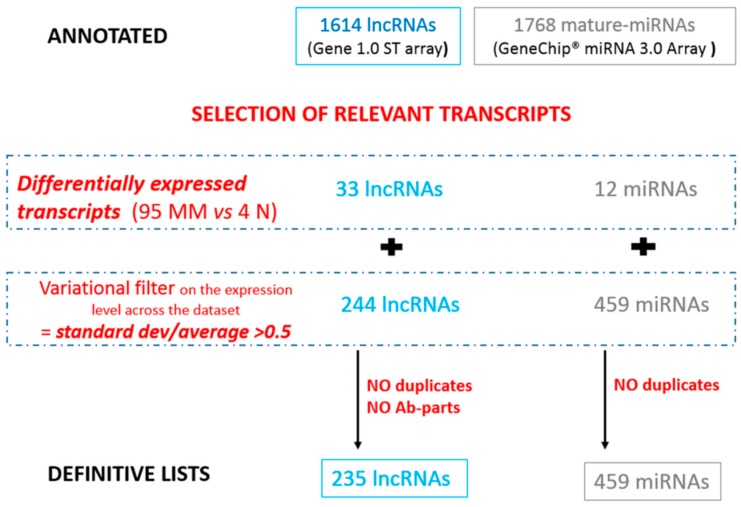
Flow chart used to select relevant lncRNAs and miRNAs for the following analyses.

The expression profiles of lncRNAs and miRNAs were investigated in a cohort of 95 MM and four normal controls. To be confident in the detection of specific lncRNAs, we applied a custom annotation pipeline that remapped the probes included in the original array to distinct lncRNAs, according to the last updated LNCipedia-v4 database genomic annotations. Such a strategy led us to investigate the expression levels of 1614 well-annotated and specific human lncRNAs. Concerning miRNAs, the arrays could detect 1768 mature miRNAs. To focus on relevant lncRNA–miRNA interactions, the full lists of detectable transcripts were subsequently shortened according to the following criteria. First, we included in each list the lncRNAs and miRNAs suggestive of a role in the pathogenesis of MM by selecting those lncRNAs and miRNAs that resulted as differentially expressed in a supervised comparison between MM and normal PCs (setting the threshold for the 90th percentile of false discovery rate to the null value). Next, based on the highly heterogeneous genetic background of MM, all lncRNAs and miRNAs fulfilling a variational filter were included. From the resulting list of lncRNAs, we excluded those located in chromosomal regions, such as 14q32, 2p, and 22q, coding for the highly variable portions of the immunoglobulin genes or for IGHV pseudogenes (named Ab-parts), whose expression is related to the highly complex transcriptional activity of such regions to achieve a polyclonal antibody repertoire. Ultimately, for further analyses, we focused on 235 lncRNAs and 459 miRNAs (Figure 1, Supplementary Materials Table S1).

#### 3.1.2. Identification of lncRNAs miRNA-Target

To identify the relationship between lncRNAs and miRNAs, we first investigated which lncRNAs could be a potential miRNA target. Two of the most common target prediction algorithms (RNA-22 and miRanda) were run on each miRNA/lncRNA from the above-defined lists, testing the miRNA sequences annotated on miRbase v20 against the LNCipedia-annotated lncRNA sequences corresponding to the fragments investigated by the probes on the array. Hence, the target prediction analysis is able to specify which of the different transcripts, if any, of each of the 235 lncRNA was targeted by any of the 459 miRNAs. We identified 12,844 lncRNA–miRNA pairs supported by both target prediction algorithms (after correction of RNA22-derived data at FDR <5% for multiple testing). Specifically, the obtained pairs represented the combination of 410 miRNAs and different transcripts from 113 lncRNAs.

#### 3.1.3. Correlation between lncRNAs and Corresponding miRNA Targets in MM

Therefore, to test the occurrence of a potential transcriptional association between lncRNAs and miRNAs, we investigated the correlations between the expression levels of each miRNA/lncRNA defined above in all the patients of the cohort. We identified 11 lncRNA–miRNA pairs for which the target relationship has been predicted and whose expression was significantly anti-correlated in our database (*q*-value < 0.05) (Table 1).

### 3.2. lncRNA and miRNA Interplay in PCL

To investigate whether the interplay of lncRNAs and miRNAs identified in MM may occur in other forms of plasma cell dyscrasia, we evaluated the correlation between the expression levels of the pairs reported in Table 1 in a cohort of 30 PCL profiled for lncRNA and miRNA expressions as previously described. Notably, lnc-MCL1-2 expression was significantly anti-correlated with those of mir-106a-5p, mir-18a-5p, and mir-18b-5p, and lnc-AGBL1-4 with mir-185-5p (Table 2).

## 4. Discussion

Like most human cancers, MM is frequently associated with an altered transcription pattern involving both protein-coding RNAs [33,34], and multiple noncoding members [5,25,35]. Recently, we reported the deregulation of lncRNAs in all major forms of plasma cell dyscrasia as compared to healthy controls; in particular, distinct lncRNA transcriptional fingerprints characterize specific MM subgroups. Furthermore, we found lncRNAs whose expression was progressively deregulated in association with more aggressive stages of the pathology, suggesting a possible role in the progression of the disease [25].

lncRNA and miRNAs have been established as key players in regulating various biological and pathological processes, such as cell-cycle progression, chromatin remodeling, gene transcription, and posttranscriptional processing; furthermore, the cross-regulation between lncRNAs and miRNAs has emerged [9,10,36].

The present study provides an unprecedented overview of the miRNA/lncRNA relationship in multiple myeloma. We have investigated in silico the lncRNA and miRNA potential connection in representative cohorts of MM and PCL patients and in normal PC controls, and identified lncRNA–miRNA pairs that may have impact in the pathology.

One of the most suggestive findings involves lnc-MCL1-2, which was both significantly anti-correlated to and predicted target of miRNAs belonging to mir-17 gene family. lnc-MCL1-2 maps less than 2 kb centromeric to the Myeloid Cell Leukemia (*MCL1*) gene: their expression levels highly correlate in our dataset (R = 0.52). In addition, members of the mir-17 family such as mir-106a-5p, mir-18a-5p, mir18b-5p, and mir-17-5p have been reported to target *MCL1* [37,38]. Based on these data, we can hypothesize a ceRNA mechanism in which lnc-MCL1-2 acts as sponge for mir-17 miRNAs family, thus regulating *MCL1* transcription. This putative ceRNA network may play a role in the pathology, as *MCL1* has been recently described as a pivotal gene for maintaining cell survival [39]. Likewise, anti-correlated expression levels of lnc-MCL1-2 and members of the mir-17 gene family were also found in PCL, thus suggesting that the importance of this interplay may be extended to other forms of PC dyscrasia.

mir-185-5p is significantly anti-correlated and predicted to target lnc-AGBL1-4, which had been found to be upregulated in MM PCs compared to normal controls [25]. As regards this pair, we established a transcriptional relationship in PCL, too. lnc-AGBL1-4 (also named LINC00052) is located less than 300 kb centromeric to the Neurotrophic Tyrosine Kinase Receptor Type 3 (*NTRK3*) gene, already known to be involved in carcinogenesis. In fact, mutated *NTRK3* gene and *ETV6–NTRK3* gene fusion following t(12;15) chromosomal translocation are associated with different types of tumors, including leukemia [40,41]. Interestingly, in hepatocellular carcinoma, lnc-AGBL1-4 has been demonstrated to regulate the expression of *NTRK3* by miR-128 and miR-485-3p to strengthen cell invasion and migration [42]. In addition, the direct targeting of mir-185-5p to *NTRK3* has been demonstrated in neuroblastoma [43]. These data suggest that, in MM and PCL, a circuitry might exist that involves lnc-AGBL1-4 and mir-185-5p ultimately leading to *NTRK3* expression regulation. Although the expression levels of lnc_AGBL1-4 and *NTRK3* were not significantly correlated in our cohort of patients, all these considerations prompt further verification of this relationship in MM.

Additional studies merit the pairs lnc-DLEU2/mir-3175, lnc-WDR73-3/mir-423-5p, and LINC00173/mir-221-3p, as all these lncRNAs were found significantly deregulated in specific molecular MM subgroups in our previous investigations [25]. Specifically, lnc-DLEU2 was upregulated in MM patients with the t(11;14) translocation and downregulated in samples with the deletion of chromosome 13q; lnc-WDR73-3 was downregulated in MM samples with the t(11;14) translocation; LINC00173 was upregulated in patients overexpressing *MAF* genes in association with the mir-221 downregulation in the same MM subgroups. This is of particular interest because the cluster miR-221/222 is strongly upregulated in a variety of solid and hematologic malignancies, and mir-221/222 inhibitors exert anti-tumor activity in vitro and in vivo in MM [44]. Understanding the interplay with LINC00173 may unravel novel mechanisms on which the modulation of mir-221 relies and might ultimately provide the rationale to investigate innovative target therapies.

## 5. Conclusions

Herein, we have provided a portrait of the most reliable lncRNA/miRNA relationships in a representative cohort of primary MM tumors by means of in silico analysis based on the integration of expression data with target predictions. Of those identified, conceivably relevant interactions for MM biology were established between lnc-MCL1-2 and mir-17 gene family, lnc-AGBL1-4 and mir-185-5p, lnc-DLEU2 and miR-3175, LINC00173 and miR-221. Overall, our study reinforce the notion that understanding the RNA crosstalk will lead to significant insights into gene regulatory networks and will contribute to the comprehension of MM patho-physiology.

## Figures and Tables

**Table 1:** Target prediction metrics of correlated lncRNA–miRNAs pairs.

lncRNA	lncRNA Chr. ^1^	miRNA	miRNA Chr. ^1^	Correlation		Target Prediction ^2^	
				Corr. Coeff.	*q*-val.	lncRNA Target Sequence from/to	*q*-val.
DLEU2:26	13q14	mir-3175	15q26	−0.35	0.0444	668–690	0.011
KLF3-AS1:2	4p14	mir-4787-5p	3p21	−0.36	0.0319	1890–1911	0.045
LINC00173:6	12q24	mir-221-3p	Xp11	−0.49	0.0003	2485–2506	0.013
LINC00173:9	12q24	mir-221-3p	Xp11	−0.49	0.0003	10737–10759	0.019
AGBL1-4	15q25.3	mir-185-5p	22q11	−0.37	0.0266	3303–3321	0.040
MCL1-2:1	1q21	mir-106a-5p	Xq26	−0.36	0.0351	5285–5306	<1E-06
MCL1-2:1	1q21	mir-18a-5p	13q31	−0.43	0.0041	4377–4397	0.043
MCL1-2:1	1q21	mir-18b-5p	Xq26	−0.35	0.0388	2903–2923	0.023
MCL1-2:1	1q21	mir-20a-5p	13q31	−0.36	0.0351	98–120	0.047
MCL1-2:1	1q21	mir-17-5p	13q31	−0.35	0.0449	184–206	0.011
WDR73-3:10	15q25.2	mir-423-5p	17q11	−0.40	0.0124	2090–2113	0.049

^1^ Chromosomal localization; **^2^** RNA22 output.

**Table 2:** lncRNAs highly correlated with miRNAs in PCL.

miRNA	lncRNA Transcript	Corr. Coeff.	*p*-val.
hsa-mir-185-5p	lnc-AGBL1-4:1	−0.451	0.012
hsa-mir-106a-5p	lnc-MCL1-2:1	−0.377	0.039
hsa-mir-18a-5p	lnc-MCL1-2:1	−0.431	0.017
hsa-mir-18b-5p	lnc-MCL1-2:1	−0.466	0.009

## Supplementary Materials

The following are available online at www.mdpi.com/2073-4425/7/12/107/s1. Table S1: List of selected relevant miRNAs and lncRNAs.
