# Supplementary material for: In Silico Characterization of miRNA and Long Non-Coding RNA Interplay in Multiple Myeloma
Source: Genes (Basel). 2016 Nov 29;7(12):107. doi: 10.3390/genes7120107 (PMC5192483; doi:10.3390/genes7120107)
Supplement: Supplementary file 1 [file genes-07-00107-s001.docx]

Supplementary Materials: In Silico Characterization of miRNA and Long Non-Coding RNA Interplay in Multiple Myeloma

Domenica Ronchetti, Martina Manzoni, Katia Todoerti, Antonino Neri and Luca Agnelli

**Table S1.** List of selected relevant miRNAs and lncRNAs.

| **Relevant miRNAs and lncRNAs** | | |
| --- | --- | --- |
| miRNA | | lncRNA |
| hsa-let-7c-5p | hsa-miR-3911 | EPB41L4A-AS1 |
| hsa-let-7e-5p | hsa-miR-3916 | GAS6-AS1 |
| hsa-let-7f-5p | hsa-miR-3917 | LINC00299 |
| hsa-let-7i-3p | hsa-miR-3921 | lnc-ADAP2-2 |
| hsa-miR-100-5p | hsa-miR-3927-3p | lnc-AE000662.92.1-1 |
| hsa-miR-106a-5p | hsa-miR-3935 | lnc-ATL3-1 |
| hsa-miR-106b-3p | hsa-miR-3937 | lnc-FAM19A5-5 |
| hsa-miR-10a-5p | hsa-miR-3940-3p | lnc-HSFY2-10 |
| hsa-miR-1180-3p | hsa-miR-3940-5p | lnc-POTEB-15 |
| hsa-miR-1182 | hsa-miR-3942-5p | lnc-RNASEH1-7 |
| hsa-miR-1184 | hsa-miR-3972 | lnc-SLC37A2-2 |
| hsa-miR-1202 | hsa-miR-422a | lnc-SNX29P2-5 |
| hsa-miR-1207-5p | hsa-miR-423-3p | lnc-STOM-7 |
| hsa-miR-1208 | hsa-miR-423-5p | lnc-TBL1Y-11 |
| hsa-miR-1224-5p | hsa-miR-424-3p | lnc-TCL1B-2 |
| hsa-miR-1225-5p | hsa-miR-425-3p | lnc-WRNIP1-36 |
| hsa-miR-1226-5p | hsa-miR-4253 | lnc-ZNF141-5 |
| hsa-miR-1228-3p | hsa-miR-4261 | RAMP2-AS1 |
| hsa-miR-1228-5p | hsa-miR-4269 | ARHGAP5-AS1 |
| hsa-miR-1231 | hsa-miR-4270 | ASB16-AS1 |
| hsa-miR-1234-3p | hsa-miR-4271 | CRYM-AS1 |
| hsa-miR-1244 | hsa-miR-4281 | CTBP1-AS2 |
| hsa-miR-1246 | hsa-miR-4284 | DLEU2 |
| hsa-miR-1247-3p | hsa-miR-4286 | DLGAP1-AS1 |
| hsa-miR-125a-3p | hsa-miR-4288 | FAM222A-AS1 |
| hsa-miR-125a-5p | hsa-miR-4298 | FBXL19-AS1 |
| hsa-miR-125b-2-3p | hsa-miR-4299 | FER1L6-AS2 |
| hsa-miR-125b-5p | hsa-miR-4304 | GAS5 |
| hsa-miR-126-3p | hsa-miR-4306 | HEXA-AS1 |
| hsa-miR-1260b | hsa-miR-4310 | ILF3-AS1 |
| hsa-miR-1263 | hsa-miR-4315 | JPX |
| hsa-miR-1266-5p | hsa-miR-4317 | KLF3-AS1 |
| hsa-miR-1268a | hsa-miR-4319 | KTN1-AS1 |
| hsa-miR-1268b | hsa-miR-4322 | LINC00173 |
| hsa-miR-1269a | hsa-miR-4327 | LINC00174 |
| hsa-miR-1269b | hsa-miR-4417 | LINC00189 |
| hsa-miR-1271-5p | hsa-miR-4423-3p | LINC00339 |
| hsa-miR-1272 | hsa-miR-4428 | LINC00461 |
| hsa-miR-1273f | hsa-miR-4429 | LINC00467 |
| hsa-miR-1275 | hsa-miR-4430 | LINC00471 |
| hsa-miR-128-3p | hsa-miR-4433a-3p | LINC00472 |
| hsa-miR-1281 | hsa-miR-4433b-5p | LINC00525 |
| hsa-miR-1287-5p | hsa-miR-4436b-5p | LINC00643 |
| hsa-miR-129-5p | hsa-miR-4437 | LINC00888 |
| hsa-miR-1291 | hsa-miR-4440 | LINC00909 |
| hsa-miR-1295a | hsa-miR-4442 | LINC00917 |
| hsa-miR-1299 | hsa-miR-4443 | LINC00969 |
| hsa-miR-1301-3p | hsa-miR-4444 | LINC01287 |
| hsa-miR-1303 | hsa-miR-4445-3p | LINC01405 |
| hsa-miR-1307-3p | hsa-miR-4448 | LINC01446 |
| hsa-miR-130a-3p | hsa-miR-4449 | LINC01602 |
| hsa-miR-130b-3p | hsa-miR-4454 | lnc-ABCB5-3 |
| hsa-miR-132-3p | hsa-miR-4455 | lnc-AC008686.1-6 |
| hsa-miR-133a-3p | hsa-miR-4458 | lnc-AC010606.1-2 |
| hsa-miR-133b | hsa-miR-4459 | lnc-AC010606.1-3 |
| hsa-miR-138-1-3p | hsa-miR-4461 | lnc-AC010606.1-5 |
| hsa-miR-138-5p | hsa-miR-4462 | lnc-AC011450.1-2 |
| hsa-miR-139-5p | hsa-miR-4463 | lnc-AC078802.1-2 |
| hsa-miR-140-3p | hsa-miR-4466 | lnc-ACTR1B-5 |
| hsa-miR-140-5p | hsa-miR-4467 | lnc-ACTR2-3 |
| hsa-miR-141-3p | hsa-miR-4476 | lnc-AGBL1-4 |
| hsa-miR-143-3p | hsa-miR-4478 | lnc-AIDA-1 |
| hsa-miR-145-5p | hsa-miR-4481 | lnc-AKR7A3-2 |
| hsa-miR-1469 | hsa-miR-4484 | lnc-AL136219.1-6 |
| hsa-miR-146a-5p | hsa-miR-4485-3p | lnc-AL592284.1-2 |
| hsa-miR-146b-5p | hsa-miR-4486 | lnc-ANGPTL1-3 |
| hsa-miR-148a-5p | hsa-miR-4487 | lnc-ANKRD18B-4 |
| hsa-miR-148b-3p | hsa-miR-4488 | lnc-ANKRD30B-7 |
| hsa-miR-149-3p | hsa-miR-4489 | lnc-ARFIP1-5 |
| hsa-miR-150-3p | hsa-miR-4490 | lnc-ARID5A-3 |
| hsa-miR-150-5p | hsa-miR-4492 | lnc-ASTN1-3 |
| hsa-miR-151a-3p | hsa-miR-4496 | lnc-ATP12A-1 |
| hsa-miR-151a-5p | hsa-miR-4497 | lnc-BAG1-2 |
| hsa-miR-151b | hsa-miR-4498 | lnc-BBS1-2 |
| hsa-miR-152-3p | hsa-miR-4499 | lnc-BCAT1-2 |
| hsa-miR-155-5p | hsa-miR-4505 | lnc-BCL2L13-1 |
| hsa-miR-1587 | hsa-miR-4507 | lnc-BRI3-1 |
| hsa-miR-15a-5p | hsa-miR-4508 | lnc-BZRAP1-1 |
| hsa-miR-17-3p | hsa-miR-4516 | lnc-C12orf50-6 |
| hsa-miR-17-5p | hsa-miR-451a | lnc-C1orf132-1 |
| hsa-miR-181a-2-3p | hsa-miR-4521 | lnc-C1QTNF9B-1 |
| hsa-miR-181a-3p | hsa-miR-4526 | lnc-C20orf173-4 |
| hsa-miR-181a-5p | hsa-miR-4529-3p | lnc-C5orf39-1 |
| hsa-miR-181b-5p | hsa-miR-4530 | lnc-C9orf86-1 |
| hsa-miR-181c-5p | hsa-miR-4532 | lnc-CHRDL2-3 |
| hsa-miR-181d-5p | hsa-miR-4534 | lnc-CNTLN-4 |
| hsa-miR-182-5p | hsa-miR-4535 | lnc-COX6C-2 |
| hsa-miR-1825 | hsa-miR-4538 | lnc-CPNE1-2 |
| hsa-miR-183-5p | hsa-miR-4539 | lnc-CYorf15A.1-2 |
| hsa-miR-184 | hsa-miR-455-3p | lnc-CYorf17-2 |
| hsa-miR-185-5p | hsa-miR-4634 | lnc-DDX58-6 |
| hsa-miR-187-5p | hsa-miR-4640-5p | lnc-DIP2A-7 |
| hsa-miR-18a-3p | hsa-miR-4646-5p | lnc-DIRAS2-6 |
| hsa-miR-18a-5p | hsa-miR-4649-5p | lnc-DPF3-3 |
| hsa-miR-18b-5p | hsa-miR-4651 | lnc-ENPP5-2 |
| hsa-miR-1908-5p | hsa-miR-4655-5p | lnc-FAM205B-1 |
| hsa-miR-1909-3p | hsa-miR-4656 | lnc-FAM22E-2 |
| hsa-miR-1909-5p | hsa-miR-4665-5p | lnc-FAM65B-3 |
| hsa-miR-191-3p | hsa-miR-4667-5p | lnc-FAM65B-7 |
| hsa-miR-1910-5p | hsa-miR-4668-5p | lnc-FAR1-7 |
| hsa-miR-1911-5p | hsa-miR-4669 | lnc-FGD3-3 |
| hsa-miR-1915-3p | hsa-miR-4673 | lnc-FGD3-5 |
| hsa-miR-192-5p | hsa-miR-4674 | lnc-GALNT7-3 |
| hsa-miR-193a-3p | hsa-miR-4685-5p | lnc-GAS1-2 |
| hsa-miR-193a-5p | hsa-miR-4687-3p | lnc-GATS-4 |
| hsa-miR-193b-3p | hsa-miR-4688 | lnc-GBP2-1 |
| hsa-miR-193b-5p | hsa-miR-4689 | lnc-GJA10-9 |
| hsa-miR-194-5p | hsa-miR-4690-5p | lnc-GPR125-7 |
| hsa-miR-195-5p | hsa-miR-4695-3p | lnc-GPR160-1 |
| hsa-miR-196b-5p | hsa-miR-4695-5p | lnc-GS1-211B7.1.1-1 |
| hsa-miR-197-3p | hsa-miR-4701-3p | lnc-HAUS5-3 |
| hsa-miR-1972 | hsa-miR-4706 | lnc-HEPH-1 |
| hsa-miR-1973 | hsa-miR-4707-5p | lnc-HGSNAT-3 |
| hsa-miR-199a-3p | hsa-miR-4708-3p | lnc-HIST1H4A-1 |
| hsa-miR-199a-5p | hsa-miR-4708-5p | lnc-HK2-2 |
| hsa-miR-199b-3p | hsa-miR-4710 | lnc-HNRNPU-1 |
| hsa-miR-19a-3p | hsa-miR-4720-5p | lnc-HSP90AA1-11 |
| hsa-miR-19b-3p | hsa-miR-4721 | lnc-IFT74-11 |
| hsa-miR-200c-3p | hsa-miR-4725-3p | lnc-IL17RA-4 |
| hsa-miR-204-3p | hsa-miR-4728-5p | lnc-INSL6-3 |
| hsa-miR-204-5p | hsa-miR-4732-5p | lnc-KATNAL2-11 |
| hsa-miR-206 | hsa-miR-4734 | lnc-KBTBD6-2 |
| hsa-miR-20a-5p | hsa-miR-4736 | lnc-KDM5D-2 |
| hsa-miR-20b-5p | hsa-miR-4739 | lnc-KIAA0317-1 |
| hsa-miR-21-3p | hsa-miR-4741 | lnc-KIAA1467-2 |
| hsa-miR-21-5p | hsa-miR-4742-5p | lnc-KIAA1755-3 |
| hsa-miR-210-3p | hsa-miR-4743-5p | lnc-KIAA1755-4 |
| hsa-miR-2110 | hsa-miR-4745-5p | lnc-LGALS14-1 |
| hsa-miR-214-3p | hsa-miR-4749-5p | lnc-LINS-1 |
| hsa-miR-219b-5p | hsa-miR-4750-5p | lnc-LIPI-4 |
| hsa-miR-22-3p | hsa-miR-4758-5p | lnc-LPA-6 |
| hsa-miR-221-3p | hsa-miR-4763-3p | lnc-LRMP-7 |
| hsa-miR-221-5p | hsa-miR-4767 | lnc-LRRC17-1 |
| hsa-miR-222-3p | hsa-miR-4773 | lnc-LRRTM4-3 |
| hsa-miR-223-3p | hsa-miR-4776-5p | lnc-LYZL2-3 |
| hsa-miR-2277-3p | hsa-miR-4783-3p | lnc-MAGEA12-3 |
| hsa-miR-2277-5p | hsa-miR-4784 | lnc-MAGOHB-1 |
| hsa-miR-2392 | hsa-miR-4785 | lnc-MARVELD3-3 |
| hsa-miR-23a-5p | hsa-miR-4787-5p | lnc-MC5R-1 |
| hsa-miR-23b-3p | hsa-miR-4793-3p | lnc-MCL1-1 |
| hsa-miR-24-2-5p | hsa-miR-4799-3p | lnc-MCL1-2 |
| hsa-miR-2467-3p | hsa-miR-4800-3p | lnc-MDGA1-3 |
| hsa-miR-25-5p | hsa-miR-4800-5p | lnc-MEIS2-2 |
| hsa-miR-26b-5p | hsa-miR-483-5p | lnc-METTL8-3 |
| hsa-miR-27a-3p | hsa-miR-484 | lnc-MRPL46.1-1 |
| hsa-miR-27a-5p | hsa-miR-486-3p | lnc-MT1A-1 |
| hsa-miR-27b-3p | hsa-miR-486-5p | lnc-MT1X-3 |
| hsa-miR-28-3p | hsa-miR-489-3p | lnc-MTMR2-4 |
| hsa-miR-28-5p | hsa-miR-491-5p | lnc-MUC15-3 |
| hsa-miR-2861 | hsa-miR-494-3p | lnc-NAPEPLD-5 |
| hsa-miR-29b-1-5p | hsa-miR-497-5p | lnc-NBN-2 |
| hsa-miR-29b-2-5p | hsa-miR-500a-3p | lnc-NFYA-1 |
| hsa-miR-29b-3p | hsa-miR-500a-5p | lnc-NMBR-7 |
| hsa-miR-29c-3p | hsa-miR-501-3p | lnc-NPHP1-1 |
| hsa-miR-29c-5p | hsa-miR-501-5p | lnc-NTNG1-2 |
| hsa-miR-30a-3p | hsa-miR-502-3p | lnc-OR10D3-3 |
| hsa-miR-30a-5p | hsa-miR-503-5p | lnc-OR2AG2-1 |
| hsa-miR-30b-5p | hsa-miR-505-5p | lnc-OR3A1-1 |
| hsa-miR-30c-5p | hsa-miR-506-3p | lnc-OR4E2-6 |
| hsa-miR-30d-5p | hsa-miR-508-5p | lnc-OR4E2-9 |
| hsa-miR-3124-5p | hsa-miR-509-3p | lnc-ORMDL2-1 |
| hsa-miR-3128 | hsa-miR-513a-5p | lnc-OTX2-6 |
| hsa-miR-3135b | hsa-miR-513b-5p | lnc-P4HA2-2 |
| hsa-miR-3136-5p | hsa-miR-513c-5p | lnc-PLEKHH2-4 |
| hsa-miR-3141 | hsa-miR-514b-5p | lnc-PLXNC1-2 |
| hsa-miR-3147 | hsa-miR-532-3p | lnc-POLR3G-1 |
| hsa-miR-3154 | hsa-miR-532-5p | lnc-POM121L2-2 |
| hsa-miR-3156-5p | hsa-miR-542-5p | lnc-POTEF-6 |
| hsa-miR-3157-3p | hsa-miR-548a-3p | lnc-PQLC2-1 |
| hsa-miR-3162-5p | hsa-miR-548ac | lnc-RBM11-5 |
| hsa-miR-3175 | hsa-miR-548ae-3p | lnc-RNASE3-1 |
| hsa-miR-3176 | hsa-miR-548aj-3p | lnc-RNF169-1 |
| hsa-miR-3178 | hsa-miR-548q | lnc-RP11-121M22.1.1-1 |
| hsa-miR-3180 | hsa-miR-548u | lnc-RP11-419C5.2.1-3 |
| hsa-miR-3180-3p | hsa-miR-548x-3p | lnc-RUSC2-2 |
| hsa-miR-3184-3p | hsa-miR-550a-5p | lnc-SCRN1-4 |
| hsa-miR-3185 | hsa-miR-551b-3p | lnc-SDCBP-2 |
| hsa-miR-3187-3p | hsa-miR-570-3p | lnc-SEC16B.1-2 |
| hsa-miR-3187-5p | hsa-miR-572 | lnc-SERHL2-4 |
| hsa-miR-3188 | hsa-miR-574-3p | lnc-SERPINB9-1 |
| hsa-miR-3195 | hsa-miR-574-5p | lnc-SERPINC1-2 |
| hsa-miR-3196 | hsa-miR-575 | lnc-SGMS1-4 |
| hsa-miR-3197 | hsa-miR-578 | lnc-SHCBP1-3 |
| hsa-miR-3201 | hsa-miR-584-5p | lnc-SOD3-14 |
| hsa-miR-320a | hsa-miR-593-5p | lnc-SORCS2-1 |
| hsa-miR-320b | hsa-miR-602 | lnc-SSX2B-5 |
| hsa-miR-320c | hsa-miR-625-5p | lnc-STRC-1 |
| hsa-miR-320d | hsa-miR-628-3p | lnc-TAF1C-1 |
| hsa-miR-320e | hsa-miR-629-3p | lnc-TAMM41-3 |
| hsa-miR-324-3p | hsa-miR-629-5p | lnc-TBX20-3 |
| hsa-miR-324-5p | hsa-miR-638 | lnc-TEX2-2 |
| hsa-miR-328-5p | hsa-miR-642b-3p | lnc-TM6SF2-1 |
| hsa-miR-330-3p | hsa-miR-650 | lnc-TMEM234-3 |
| hsa-miR-331-3p | hsa-miR-652-3p | lnc-TNFRSF13B-5 |
| hsa-miR-331-5p | hsa-miR-660-5p | lnc-TOMM7-1 |
| hsa-miR-335-5p | hsa-miR-663a | lnc-TOR1AIP2-5 |
| hsa-miR-339-3p | hsa-miR-663b | lnc-TPTE-5 |
| hsa-miR-339-5p | hsa-miR-664a-5p | lnc-TSN-3 |
| hsa-miR-342-3p | hsa-miR-665 | lnc-UHRF1-1 |
| hsa-miR-342-5p | hsa-miR-671-5p | lnc-ULBP3-3 |
| hsa-miR-345-5p | hsa-miR-708-5p | lnc-UNC13B-2 |
| hsa-miR-346 | hsa-miR-744-5p | lnc-UQCRFS1-3 |
| hsa-miR-34a-5p | hsa-miR-762 | lnc-WDR73-3 |
| hsa-miR-3591-3p | hsa-miR-766-3p | lnc-ZNF124-2 |
| hsa-miR-3607-5p | hsa-miR-767-5p | lnc-ZNF131-1 |
| hsa-miR-3609 | hsa-miR-769-5p | lnc-ZNF22-2 |
| hsa-miR-361-3p | hsa-miR-874-3p | lnc-ZNF252-2 |
| hsa-miR-3613-3p | hsa-miR-877-5p | lnc-ZNF300-1 |
| hsa-miR-3613-5p | hsa-miR-885-3p | lnc-ZNF583-4 |
| hsa-miR-3615 | hsa-miR-885-5p | lnc-ZNF595-1 |
| hsa-miR-3619-5p | hsa-miR-9-3p | lnc-ZNF721-1 |
| hsa-miR-362-5p | hsa-miR-92a-1-5p | lnc-ZNF737-3 |
| hsa-miR-3621 | hsa-miR-92a-3p | lnc-ZNF8-4 |
| hsa-miR-363-3p | hsa-miR-92b-3p | MIR155HG |
| hsa-miR-3646 | hsa-miR-92b-5p | MIR17HG |
| hsa-miR-3648 | hsa-miR-93-3p | MIR222HG |
| hsa-miR-3651 | hsa-miR-93-5p | MIR22HG |
| hsa-miR-3652 | hsa-miR-933 | MIR99AHG |
| hsa-miR-3656 | hsa-miR-935 | PAX8-AS1 |
| hsa-miR-365a-5p | hsa-miR-936 | RHPN1-AS1 |
| hsa-miR-3663-3p | hsa-miR-939-5p | SNHG1 |
| hsa-miR-3665 | hsa-miR-940 | SNHG10 |
| hsa-miR-3679-5p | hsa-miR-99a-5p | SNHG15 |
| hsa-miR-3687 | hsa-miR-99b-5p | SNHG16 |
| hsa-miR-371b-5p |  | SNHG17 |
| hsa-miR-375 |  | SNHG22 |
| hsa-miR-378a-3p |  | SNHG25 |
| hsa-miR-378a-5p |  | SNHG5 |
| hsa-miR-378c |  | SNHG8 |
| hsa-miR-378d |  | TCL6 |
| hsa-miR-378f |  | TFAP2A-AS1 |
| hsa-miR-378g |  | TP73-AS1 |
| hsa-miR-378h |  | TTTY10 |
| hsa-miR-378i |  | TTTY13 |
| hsa-miR-3910 |  | ZNF667-AS1 |
